# Pattern of Radiographic and Sonographic Findings of Adult Patients Presented with Shoulder Pain at Tikur Anbessa Hospital, Addis Ababa, Ethiopia

**DOI:** 10.4314/ejhs.v33i6.9

**Published:** 2023-11

**Authors:** Bemnet Taye Gebregiorgis, Moges Zenebe Wegayehu

**Affiliations:** 1 Department of Radiology,College of Health Sciences,AAU,Addis Ababa, Ethiopia

**Keywords:** Shoulder Ultrasound, Shoulder Radiograph, Rotator Cuff, Musculoskeletal Imaging

## Abstract

**Background:**

Shoulder pain is one of the most common presentations in the orthopedic clinic. Multiple factors have been found to cause shoulder pain. Radiographs and ultrasound are widely available, relatively cheap modalities in assessing shoulder pain. The aim of this study is to assess the radiographic and sonographic imaging patterns of shoulder pain.

**Methods:**

A descriptive prospective cross-sectional hospital-based study was conducted at the Department of Radiology of Tikur Anbessa Specialized Hospital among patients with shoulder pain that came for imaging from August 2021-January 2022.

**Result:**

Of the 73 patients with shoulder pain included in the study, 67% were females while 33% males. The mean age was 51.7 years. Radiographs found pathology in 53% of the cases. Acromioclavicular joint osteoarthritis, greater tuberosity degenerative changes, rotator cuff calcification were common radiographic findings. Ultrasound detected pathologies in 87% of the cases. The common pathologies were rotator cuff pathologies, biceps tendon pathologies, acromioclavicular joint degeneration, greater tuberosity degenerative changes, subacromial subdeltoid bursitis, and adhesive capsulitis. There was a significant association between greater tuberocity degenerative changes and supraspinatous pathologies with age, greater tuberocity degenerative changes with supraspinatous pathologies, acromiohumeral distance of <7mm with supraspinatous pathology.

**Conclusion:**

Radiographs and ultrasound are valuable imaging modalities for shoulder pain. Low acromiohumeral interval, greater tuberosity degenerative changes, and acromioclavicular joint osteoarthritis are associated with rotator cuff tears. Rotator cuff pathologies are the most common pathologies observed in ultrasound. We recommend ultrasound to be second step after radiograph due to its low cost and wide availability.

## Introduction

Shoulder pain is one of the most common presentations in the orthopedic clinic, prevalence ranging from 2.4-26% (1). Chronic shoulder pain without antecedent trauma is a common medical morbidity in middle aged and elderly populations (2). Some of the factors that have correlation with shoulder pain were age greater than 60 years, female sex, sitting with cervical spine flexion, repetitive physical activity that involves the shoulder including repetitive overhead activities (3).

The glenohumeral and acromioclavicular joint (ACJ) are the articular structures around the shoulder. The glenohumeral joint is ball and socket type of synovial joint with the synovium lining the internal joint capsule extending to the long head of biceps tendon (LBT). Many structures comprise the shoulder joint including the rotator cuff tendons, superior, middle and inferior glenohumeral ligaments, coracohumeral ligament, and the circumferential labrum surrounding the glenoid fossa (1).

The most common pathologies are rotator cuff pathologies followed by ACJ arthritis, LBT tendinitis, and subacromial–subdeltoid (SA-SD) bursitis. A study done in Kenya (D Mang'Oka etal) and a similar observation in another study (Li Jiang et-al) found subacromial disorders including rotator cuff tendinosis, calcific tendinosis, rotator cuff tear, and subdeltoid bursitis accounting for the majority of shoulder pathologies (4,5).

Subacromial impingment syndrome is the most common cause of shoulder pain. It results from impingment of the supraspinatous tendon and bursa under the coraco-acromial arch. The coraco-acromial arch structures including the acromion, coraco-acromial ligament and ACJ abnormalities can cause irritation of the supraspinatous tendon and overlying bursa. This results in a spectrum of abnormalities including bursitis, calcific tendinosis, supraspinatous tear, and different degrees of rotator cuff tendinopathy. The ACJ and type of acromion can be evaluated by radiograph and MRI. MRI and ultrasound (US) can visualize and demonstrate different abnormalities of the supraspinatous tendon. Dynamic ultrasound evaluation is also very important in establishing the diagnosis (6,7). Ultrasound and MRI can show fluid in the subacromial bursa, calcification of the tendons, ACJ degenerative changes, rotator cuff tendinosis, partial or full thickness rotator cuff tears, synovial thickening, and biceps tenosynovitis (8).

The first imaging modality that should be done is radiography. There are different radiographic projections in evaluation of shoulder pathologies including anterioposterior (AP), external or internal rotation, axillary, scapular Y views, supraspinatous outlet, and oblique views. The basic views are AP view and grashey view. These views aid in visualization of ACJ osteophytes, type of acromion, and os-acromiale. We can also obtain acromiohumeral distance (AHD) (normal being >7mm), greater tuberosity cortical irregularities, sclerosis, and cysts (6). Due to limitation of radiography in evaluating soft tissue structures around the shoulder, additional imaging modality is important. There are many researches and views on whether ultrasound or MRI should be the next imaging modality. However, there are good evidences to do either imaging modalities. MRI arthrography is recommended for labral tears. MRI has the disadvantages of high cost, longer time, and contraindications like metallic implants and pacemakers. Although ultrasound is cost-effective, it needs expertise and is highly operator-dependent (6).

Proper diagnosis of shoulder abnormalities is essential as the management differs from medical, physiotherapy, and surgical choices. The first step in evaluation of patients presenting with shoulder pain is history-taking and physical examination with multiple clinical tests. However, physical examination lacks sensitivity in diagnosis of these conditions (6,7).

Ultrasound has an important role in diagnosis of rotator cuff tears. It was reported that ultrasound has better sensitivity and specificity in diagnosing full thickness tears, which is comparable to conventional MRI. Ultrasound has sensitivity of 95% and specificity of 96% in diagnosing full thickness tears, while it has sensitivity of 72% and specificity of 93% in diagnosing partial thickness tears. Ultrasound is relatively cheap, widely available, faster imaging modality that does not have contra indications. Due to its cost-effectiveness, use of ultrasound is advocated in secondary care settings and MRI preserved for other soft tissue structures that ultrasound cannot assess such as labral pathologies (7).

Currently, in Ethiopia, ultrasound is becoming widely available. Especially, we are getting access to high resolution ultrasound machines, which are very helpful in evaluation of musculoskeletal examinations including shoulder scans. Still, there is lack of expert doing musculoskeletal ultrasound, and also clinicians do not have adequate knowledge regarding the role of ultrasound in shoulder pathology evaluation. We do not have local data regarding the role of imaging studies in the assessment of shoulder pathologies. By assessing the radiographic and sonographic imaging patterns of shoulder pain patients, the study will help the medical community understand the role of radiography and ultrasound for evaluation of shoulder pathologies.

## Materials and Methods

**Study design and setting**: The study was conducted at radiology department of Tikur Anbessa Specialized Hospital, Addis Ababa University. The hospital is located in Addis Ababa, Ethiopia. It is the largest referral as well as the main teaching hospital. The hospital provides a tertiary level referral treatment with 24hrs emergency services. The hospital has more than 700 beds and gives diagnostic and treatment services for about 370,000-400,000 patients per year. The Department of Radiology has currently more than 30 staff members, and of those, five are musculoskeletal radiology staff. The department is involved in academic and clinical activities. The study was conducted from August, 2021-January 2022.

**Study design and population**: A descriptive prospective cross-sectional hospital-based study was conducted at the Radiology Department of Tikur Anbessa Hospital. The source population was all patients with shoulder pain who were being evaluated during the study period. The study population was all patients referred to the Radiology Department for shoulder radiograph and shoulder ultrasound. Patients with shoulder pain who were older than 18 years of age and agreed for the study by formal informed consent were included in the study, as the Musculoskeletal Imaging Department in the hospital receives adult patients. Patients with history of trauma were excluded. Non-probability consecutive sampling was used as all patients with shoulder pain sent for image studies to Tikur Anbessa Hospital during the study period were included.

**Data collection procedures**: AP radiograph of shoulder in neutral position was obtained. Ultrasound was performed by musculoskeletal radiology fellow using high resolution linear probe on Mindray machine. The study used the European Society of musculoskeletal radiology scanning protocol (9). Patients were sitted on a couch and systematic ultrasound of shoulder joint was performed. The interpretation of the radiographs and the sonographic findings was done by a musculoskeletal radiology fellow. Results were recorded on standardized questionnaire. The data was checked for clarity and completeness before it was analyzed by using SPSS version 22.0 computer software.

**Ethical considerations**: Ethical clearance was obtained from the Department of Radiology Research and Ethics Committee before the data collection process. The participants were informed. They were given the right to refuse. Data was kept confidential by not recording names of patients.

## Results

**Figure 1:**
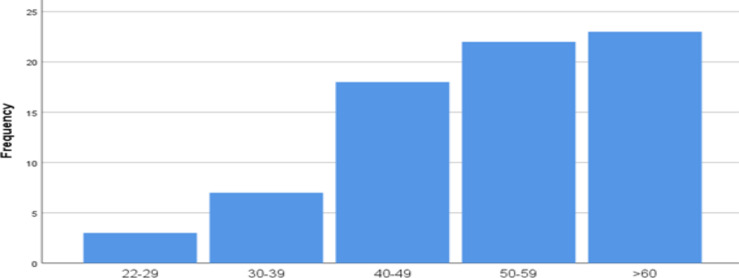
Age category distribution of patients that presented with shoulder pain in Tikur Anbessa Hospital, Addis Ababa, Ethiopia, From August 2021-January 2022

**Socio-Demographic Characteristics**: A total of 73 patients with shoulder pain were included in the study. Out of the 73 patients 49 were females, accounting for 67%, and 24 were males, accounting for 33% of the patients. The mean age was 51.7 years with standard deviation of +/-12.2. The youngest participant was 22 years while the oldest was 79year old. According to age group, young (less than 45 years) account for 24.3%, middle age (between 45 and 60 years) account for 43.2%, and old age (above 60 years) account for 31.1% of the cases (Figure 1).

**Findings on radiograph**: All the 73 patients had proper AP radiographs of the shoulder. Of these, 39 had abnormal radiographs while 34 were normal. This suggests that radiographs have 53% sensitivity in detecting pathologies.

**Figure 2:**
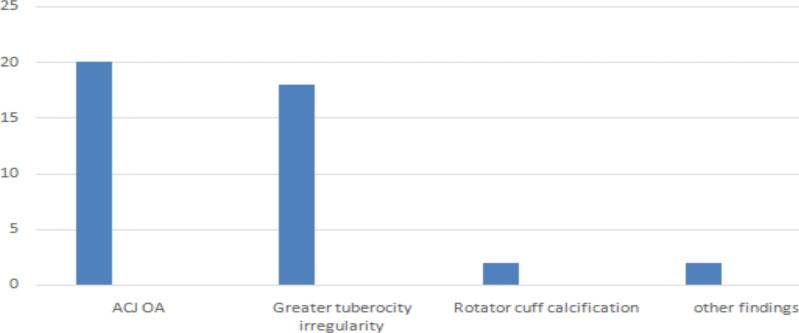
Overall distribution of Radiographic findings in patients that presented with shoulder pain Tikur Anbessa Hospital, Addis Abeba, Ethiopia, from August 2021-January 2022

The most common radiologic finding was ACJ osteoarthritis (OA). Twenty patients (27%) had ACJ degenerative changes on radiograph, followed by greater tuberosity degenerative changes in 18 patients (24.3%); 2 patients (3%) had rotator cuff calcification (calcific tendinitis) while 1 patient had clavicle fracture with ACJ dislocation and 1 patient had generalized osteopenia. There was no patient with glenohumeral arthritis in our study (Figure 2). The mean acromiohumeral distance (AHD) was 10.4mm, ranging from 1.9mm to 15mm.

**Figure 3:**
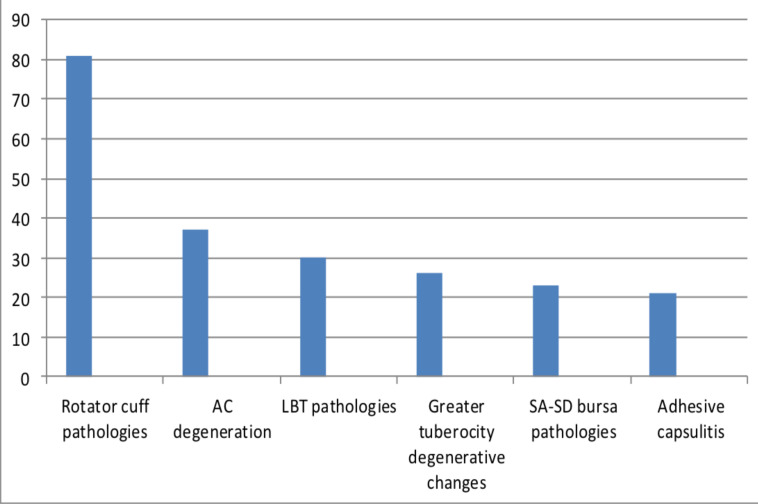
Overall distribution of Sonographic findings in patients that presented with shoulder pain Tikur Anbessa Hospital, Addis Ababa, Ethiopia, from August 2021-January 2022

**Findings on ultrasound**: Shoulder pathologies were found in 64(87%) of the cases, and no sonographic finding was found on 9 cases (13%). This suggests that ultrasound has 87% sensitivity in detecting pathologies. A total of 221 pathologies were visualized. Of those, rotator cuff pathologies predominate, accounting for 37% (81) of the findings, followed by ACJ degeneration (17%) (37 cases), LBT pathologies (14%) (30cases), greater tuberosity degenerative changes (12%) (26 cases), SA-SD bursitits (10%/(23 cases), and adhesive caspulitis (9%) (21 cases).One patient had pyomyositis, 1 had clavicle fracture and 1 had intramuscular lipoma in the deltoid muscle (Figure 3).

**Figure 4:**
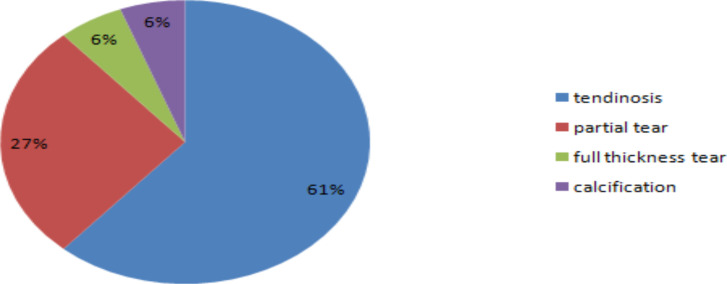
Pie Chart showing the distribution of supraspinatous pathologies in patients presenting with shoulder pain in Tikur Anbessa Hospital, Addis Ababa Ethiopia From August 2021-January 2022

**Rotator cuff sonographic findings**: Among the rotator cuff pathologies, supraspinatous tendon lesions predominated. There were 49 cases with supraspinatous lesions, 26 cases with infraspinatous lesions, and 6 cases with subscapularies lesions. Tendinosis accounted for 62% of the supraspinatous tendon pathologies, followed by partial tear, accounting for 27%, full thickness tear (5.5%) and calcification (5.5%). There were 4 cases with supraspinatous muscle atrophy/fatty replacement, and all were seen in patients with full thickness supraspinatous tendon tear (Figure 4). There were 23 cases with infraspinatous tendinopathy and 3 patients with partial tear of the infraspinatous tendon. All the 6 lesions of the subscapularies tendon were tendinopathies.

**Associations between variables**: There was a significant association between age and degenerative changes in the greater tuberosity with P-value of 0.017. The presence of degenerative changes in the greater tuberocity increases with age. There was also a significant association between greater tuberocity degenerative changes on ultrasound and supraspinatous tendon lesions with P-value of <0.001. The sensitivity of greater tuberosity irregularies for supraspinatous tear was 79.2%, and specificity was 86% (Table 1). The sensitivity of greater tuberosity irregularities for any supraspinatous pathology (tear and tendinopathy) was 51% and specificity was 96%. There was a significant association between age and supraspinatous pathologies with P-value of <0.001. Seventy seven percent of patients with supraspinatous pathologies were greater than 50 years of age, and 92% of the patients, who were older than 60 years, had supraspinatous tendon lesion on ultrasound. Age was also correlated with ACJ degeneration with P-value of 0.02 and greater tuberosity irregularities with P-value of 0.001. There was a significant association between supraspinatous tendon pathologies and AHD. AHD of <7mm had association with presence of supraspinatous tendon lesion with P-value of 0.07. There was a significant association between greater tuberosity degenerative changes on radiograph and supraspinatous tendon pathologies on ultrasound with P-value <0.001, OR 1.774(1.406-2.239). Another significant association was detected between ACJ degenerative changes on radiograph and supraspinatous tendon pathologies on ultrasound with P-value of 0.02.

**Table 1:** Association between greater tuberosity degenerative changes and supraspinatous lesions in patients with shoulder pain at TikurAnbessa Hospital, Addis Abeba, Ethiopia, from August 2021-January 2022

Greater tuberosity irregularities	Presence of supraspinatous tear	No evidence of supraspiantous tear (Normal or tendinosis)	Total
**Present**	19(79.2%)	7(14.3%)	26(35.6%)
**Absent**	5(20.8%)	42(85.7%)	47(64.4%)
**Total**	24(32.8%)	49(67.2%)	73(100%)

## Discussion

Shoulder pain is one of the most common presentations in the orthopedic clinic, prevalence ranging from 2.4-26% (1). This study is one of its kinds in evaluation of the role of ultrasound and radiograph in evaluation of shoulder pain in Ethiopia. The study reviewed 73 patients with shoulder pain that came to Tikur Anbessa Specialized Hospital. The study showed a higher prevalence of shoulder pain in the female population (67%). Multiple studies show similar gender prevalence. D.Mango'oka etal also showed female prevalence with 3:2 female to male ratio, which is similar to our study. Li Jiang etal also arrived at similar findings. This can be explained by the increased prevalence of adhesive capsulitis in females (12). We recommend well-structured studies to explore why shoulder pain is more common in females. The mean age of our participants was 51.7 years, and the age ranged from 22 to 79 years. In our study, we also observed that middle age and old age groups accounted for over 3/4^th^ of the cases. D.Mango etal and Li Jiang etal also showed increased prevalence with age (4,5).

Radiographs showed abnormality in 53% of the cases. The commonest findings were ACJ osteo-arthritis and degenerative changes on greater tuberosity. Few cases of calcific tendinosis were seen. Cadogan etal also showed that the predominant findings in shoulder radiographs were degenerative changes and calcific tendinosis (16). A study done in Kenya also showed that half of the radiographs were normal, and degenerative changes were the main findings in abnormal radiographs and fewer cases of calcific tendinitis observed in their study (5).

The study also showed that there was a significant association between age and greater tuberosity degenerative changes with P-value of 0.017. Increasing radiographic findings were observed as age increases. D. Mango oka also showed similar findings (5).

All the patients with abnormal radiographic findings had abnormalities on ultrasound. From the 34 normal radiographs, 25 of them had findings on ultrasound. The most common ultrasound findings were rotator cuff pathologies, followed by ACJ degenerative changes, followed by LBT pathologies. Other common findings were SA-SD bursitis and adhesive capsulitis. Rotator cuff pathologies were predominant sonographic findings in a study done conducted in China (Li Jiang etal) (4). A study done in Kenya (D.Mango etal) also showed that 42% pathologies were rotator cuff pathologies (5). In Cadogan's study, 50% of the lesions were rotator cuff pathologies (10).

Among the rotator cuff pathologies, supraspinatous pathologies were predominant. From the supraspinatous pathologies, tendinopathy was commonly observed, followed by partial tear and complete tear. All the complete tears in our study were associated with supraspinatous muscle atrophy. Tendon calcifications accounted for 5.5% of the cases. Cadogan etal showed that 85% of rotator cuff pathologies had supraspinatous component. D, Mango etal also showed that supraspinatous pathology was observed in 60% of the cases (5,10). F. Zheng etal showed that calcfiic tendinosis was observed in the majority (54%), followed by tendinopathy and tear (11). In cadogan's study, tears were the most common pathology of supraspinatous, and calcifications were seen in 39% of cases (10). In our study, however, calcifications were lower, similar to the lower finding of rotator cuff calcification in a Kenyan study (5). Epidemiological factors might be responsible for such discrepancies in different regions.

There was a significant association between greater tuberosity degenerative changes on ultrasound and supraspinatous tendon lesions with P-value of <0.001. Greater tuberosity degenerative changes had sensitivity of 79% and Specificity of 86% to detect the presence of supraspinatous tear, and 51% sensitivity and 96% specificity to detect supraspinatous pathologies. Similar findings were shown in the Kenyan study, which revealed that greater tuberosity changes had sensitivity of 75% and specificity of 92% for supraspinatous tear (5). Age had association with supraspinatous pathologies in our study with P-value of <0.001. Three fourth of supraspinatous pathologies were observed in patients greater than 50 years of age and 92% of the patientswho were above 60 years, had at least one supraspinatous pathology on ultrasound. F. Zheng etal also found that age was associated with detection of rotator cuff disorder with a significant increase in the disorders with increasing age (11).

Finally, the study tried to evaluate the findings in shoulder radiographs and findings on shoulder ultrasound. The study observed that lower AHD, greater tuberosity degenerative changes, and ACJ OA had a significant association with rotator cuff pathologies. AHD of <7mm had a significant association with supraspinatous lesion with P-value of <0.07. D .Mango'ok etal also found that all of the participants with AHD <7mm had some form of supraspinatous tear. This study also found that there was a significant association between greater tuberosity degenerative changes and AC joint OA with rotator cuff tears (5).

In Conclusion, in patients with shoulder pain radiographs and ultrasound are valuable imaging modalities. Significant number of the radiographs taken were normal, but it is recommended as an initial imaging modality. Things to emphasize on radiographs include presence of degenerative changes on ACJ, degenerative changes in the greater tuberosity, calcific tendinitis, and AHD. Sonographic findings were observed on most patients presenting with shoulder pain. Rotator cuff pathologies were the most common pathologies observed in ultrasound. In our setup, we recommend ultrasound to be second step after radiograph due to its low cost and wide availability.
